# Forecasting the evolution of fast-changing transportation networks using machine learning

**DOI:** 10.1038/s41467-022-31911-2

**Published:** 2022-07-22

**Authors:** Weihua Lei, Luiz G. A. Alves, Luís A. Nunes Amaral

**Affiliations:** 1 Department of Physics and Astronomy, Northwestern University, Evanston, IL 60208 USA; 2 Department of Chemical and Biological Engineering, Northwestern University, Evanston, IL 60208 USA; 3 Northwestern Institute on Complex Systems (NICO), Northwestern University, Evanston, IL 60208 USA

**Keywords:** Energy science and technology, Complex networks

## Abstract

Transportation networks play a critical role in human mobility and the exchange of goods, but they are also the primary vehicles for the worldwide spread of infections, and account for a significant fraction of *CO*_2_ emissions. We investigate the edge removal dynamics of two mature but fast-changing transportation networks: the Brazilian domestic bus transportation network and the U.S. domestic air transportation network. We use machine learning approaches to predict edge removal on a monthly time scale and find that models trained on data for a given month predict edge removals for the same month with high accuracy. For the air transportation network, we also find that models trained for a given month are still accurate for other months even in the presence of external shocks. We take advantage of this approach to forecast the impact of a hypothetical dramatic reduction in the scale of the U.S. air transportation network as a result of policies to reduce *CO*_2_ emissions. Our forecasting approach could be helpful in building scenarios for planning future infrastructure.

## Abstract (web summary)

Transportation networks undergo permanent changes influenced by a variety of human-induced and natural factors. The authors propose here a machine learning framework for prediction of connections removal that could be useful in building scenarios for transportation infrastructure needs.

## Introduction

Transportation networks are critical infrastructures and one of the foundations of modern globalized societies. The air transportation network alone is responsible for the mobility of millions of people every day across the world^1,2^. However, transportation networks are also responsible, indirectly, for the propagation of diseases, such as influenza and, recently, severe acute respiratory syndrome coronavirus 2 (SARS-CoV-2)^3–5^. In addition to their role in enabling pandemics, transportation is also a significant contributor to greenhouse gas emissions, accounting for about 29% of the total U.S. greenhouse gas emissions and 14% of the total global greenhouse gas emissions^6,7^. Among all transportation sectors, air transportation contributes 9% of U.S. greenhouse gas emissions and 10.6% of global greenhouse gas emissions^6,7^. Even more concerning, at a time when global greenhouse gas emissions must be reduced, emissions from the transportation sector are on the rise^8,9^. However, as the consequences of climate change become inescapable^9^, it is inevitable that dramatic changes in how transportation networks are organized will occur^10^. Thus, it is crucial for planners to be able to forecast how transportation networks could evolve in the coming decades.

The study of connection dynamics in networked systems, including transportation networks, has yielded significant insights^11–15^. However, the study of the temporal dynamics of the edges in transportation networks remains underdeveloped. A significant challenge for transportation networks is that their edge dynamics are the outcome of concurrent actions of businesses motivated by competition and profit, governments motivated by national interests, and historical contingencies.

Recently, machine learning (ML) approaches have been successfully applied in the study of human mobility^16,17^, sustainability of transportation infrastructure^18^, and the impact of COVID-19 on gasoline demands^19^. Here, we take a similar approach to probe the dynamics of the edges in transportation networks. For mature transportation networks, structure changes are primarily due to the addition and removal of edges, the addition and removal of nodes being much less significant. In the past, the addition of edges has been studied mainly in the context of missing link prediction^20,21^ and network growth models^22^, whereas the removal of edges has been studied in contexts, such as network percolation^23,24^, attack and error tolerance^25^, dismantling strategies^26^, catastrophic failures^27^, synchronization and phase-transitions^28^, pruning processes based on removal of underutilized links^29^, and cascading failures^30^, to name a few. However, edge removals in real-world temporal networks do not grow unbound as in percolation or dismantling processes and the mechanism determining the removal of edges is not well understood. To address this knowledge gap and because of the practical implications of the problem, we apply machine learning algorithms to the challenge of predicting edge removals on transportation networks.

We investigate the edge dynamics of two large mature but fast-changing transportation networks: the Brazilian inter-cities bus transportation network (Brazil Bus net)^31,32^ and the U.S. domestic air transportation network (U.S. Air net)^33^. We do not consider here rail transportation networks because they tend to change very slowly. Using ML algorithms to classify edges by their topological properties, we find statistically significant differences between features of edges retained and features of edges removed. Further, we develop an ML model that enables us to forecast removed edges. We also test the robustness of our model to large external shocks, such as COVID-19 travel restrictions. We use this model to simulate the effect of a reduction in the number of connections in the U.S. domestic air transportation network and discuss the implications of our findings on building alternative scenarios for planning future infrastructure.

## Results

### Transportation networks

We collected data for the Brazilian inter-city bus transportation network (Brazil Bus net) and the United States domestic air transportation network (U.S. Air net) at a monthly temporal resolution. In the Brazil Bus net, the nodes represent cities with bus stops on a bus route. An undirected edge ${e}_{ij}^{m}$ connects nodes *i* and *j* if there is at least one bus route connecting them at some point during the month *m*. The number of buses during the month *m* is used as the weight for edge ${e}_{ij}^{m}$. We construct both a weighted and an unweighted undirected temporal network {*G*_1_ → *G*_2_ → . . . → *G*_*T*_}, where *G*_*m*_ represents the network snapshot constructed with data from month *m*.

**Fig. 1:**
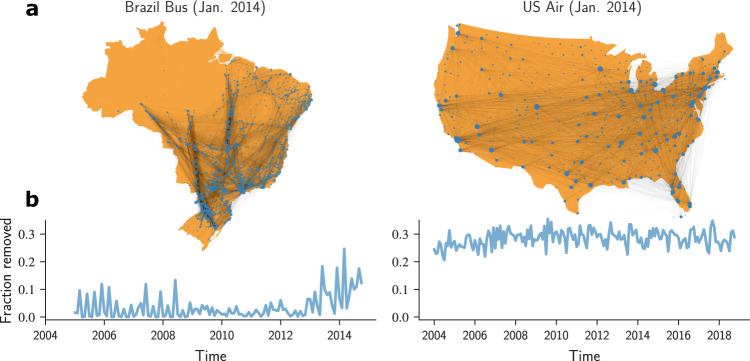
Edge dynamics in two countrywide transportation networks. **a** January 2014 snapshots of the Brazilian inter-city bus transportation network and the United States domestic air transportation network (only mainland shown). The size of each circle is proportional to the degree of the node representing the city at that location. **b** Fraction of edges removed monthly for the two networks. The maps were generated using the Python package *cartopy* version 0.20.0.

In the U.S. Air net, the nodes represent U.S. cities with airports. An edge indicates that at least one airline directly connected the two cities during the monthly observation window (Fig. 1a). In the weighted network, the weight of an edge is the number of flights during the monthly observation window. Figure 1b shows that a significant fraction of existing edges is removed from the network from one snapshot to another.

### Machine learning prediction

We formulate the question of how to predict which edges will be removed as a supervised classification problem. In a network snapshot *G*_*m*_, we assign to edges one of three states: ‘added’, ‘retained’, or ‘removed’. Added edges were not present at the beginning of the monthly observation window but are present at the end. Retained edges are present at the beginning and end of the monthly observation window. Removed edges are present at the beginning but not at the end of the monthly observation window. To achieve the goal of predicting removed edges, we only need to consider edges already present in *G*_*m*_. Those edges can only be ‘retained’ or ‘removed’. Therefore, our problem is reduced to a binary classification where the task is to determine if an edge is retained or removed in a given snapshot. The features of an edge can be extracted from *G*_*m*_ and represented as a feature vector ${{{{{{{{\bf{X}}}}}}}}}_{ij}^{m}$. Thus, we can write the probability of an edge ${e}_{ij}^{m}$ being removed as:

1 $${{{{{{{\bf{Prob}}}}}}}}\left({e}_{ij}^{m}=removed\right)=f\left({{{{{{{{\bf{X}}}}}}}}}_{ij}^{m}\right).$$

**Fig. 2:**
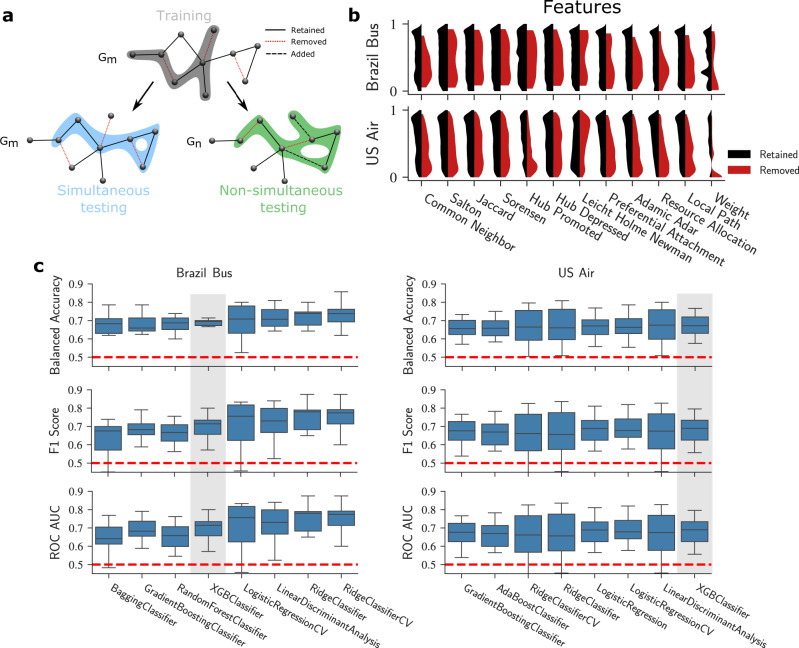
Performance of machine learning models for predicting retained and removed edges in a snapshot of a transportation network. **a** Illustration of the process for creating training and testing sets in a transportation network. Edges shown in black are retained during the observation window, whereas edges shown in red are removed. We select a fraction of edges for inclusion in the training set (grey-shaded subgraph in the figure). We identify a simultaneous testing set (blue-shaded subgraph) by considering all edges that were present at month *G*_*m*_ but were not included in the training set. Additionally, we identify a non-simultaneous testing set (green-shaded subgraph includes edges in dashed bold line that are added after the training graph) by considering all edges that exist at snapshot *G*_*n*_ where *n* > *m*, and that were not included in the training set. **b** We calculate a broad range of features for each edge and compare the distributions of said features for retained and removed sets of edges in the training set. For clarity, feature values are transformed into the range from zero to one using their 10 quantiles. It is visually apparent, for most features, that the distributions for retained and removed edges are different. **c** We evaluate the performance of 27 common supervised classification algorithms using tenfold cross-validation. We show box plots of the estimated model balanced accuracies, F1 scores, and area under the receiver operating characteristic curve (ROC-AUC) for the Brazil Bus net and the U.S. Air net. We order the algorithms by their average balanced accuracy for each network and show results for the top 8 algorithms. In the following, we focus on one of those algorithms, XGBClassifier, because it has the lowest error variance among high-performing algorithms.

To test our hypothesis that removed edges are significantly different in their topological features from those of retained edges, we randomly select 70% of retained and removed edges in a selected snapshot for inclusion in the training set. As illustrated in Fig. 2a, if a model is trained with *G*_*m*_, one can perform two different tests depending on whether the testing edges are selected from the same snapshot as the training edges. In the simultaneous test, we test on edges from the same snapshot as the training edges. In the non-simultaneous test, we test on edges from snapshots that come after the training snapshot. This test evaluates the similarity of removal dynamics for different snapshots.

### Features

Numerous features could be used to characterize an edge in a network. In much of the literature, it is assumed that edge weights are not available^20^. Thus we separately study the impacts of edge weight and edge topological features on the predictability of edge removals. We consider a subset of possible unweighted topological features used widely in the link prediction literature (Table 1). Most are local properties^34^. Therefore, one could make predictions even without knowing the structure of the entire network.

**Table 1:** Considered features: Γ_*i*_ refers to the set of neighbors of node *i*. *k*_*i*_ = ∣Γ_*i*_∣ is the degree of node *i*^40^.

Feature	Definition	Description
Common Neighbors (CN)	$\left|{{{\Gamma }}}_{i}\cap {{{\Gamma }}}_{j}\right|$	The number of common neighbors of nodes *i* and *j*
Salton Index (SA)	$\frac{\left|{{{\Gamma }}}_{i}\cap {{{\Gamma }}}_{j}\right|}{\sqrt{{k}_{i}\times {k}_{j}}}$	The number of common neighbors normalized by the geometric average degree of both nodes
Jaccard Index (JA)	$\frac{\left|{{{\Gamma }}}_{i}\cap {{{\Gamma }}}_{j}\right|}{\left|{{{\Gamma }}}_{i}\cup {{{\Gamma }}}_{j}\right|}$	The number of common neighbors normalized by the union of neighbors of both nodes
Sørensen Index (SO)	$\frac{2\left|{{{\Gamma }}}_{i}\cap {{{\Gamma }}}_{j}\right|}{{k}_{i}+{k}_{j}}$	The number of common neighbors normalized by the average degree of the two nodes
Hub Promoted Index (HPI)	$\frac{\left|{{{\Gamma }}}_{i}\cap {{{\Gamma }}}_{j}\right|}{min({k}_{i},{k}_{j})}$	The number of common neighbors normalized by the smaller degree of the two nodes
Hub Depressed Index (HDI)	$\frac{\left|{{{\Gamma }}}_{i}\cap {{{\Gamma }}}_{j}\right|}{max({k}_{i},{k}_{j})}$	The number of common neighbors normalized by the larger degree of the two nodes
Leicht-Holme-Newman Index (LHNI)	$\frac{\left|{{{\Gamma }}}_{i}\cap {{{\Gamma }}}_{j}\right|}{{k}_{i}\times {k}_{j}}$	The number of common neighbors normalized by the product of degrees of the two nodes
Preferential Attachment Index (PA)	*k*_*i*_ × *k*_*j*_	The product of the degrees of the two nodes
Adamic-Adar Index (AA)	${\sum }_{{w}_{n}\in {{{\Gamma }}}_{i}\cap {{{\Gamma }}}_{j}}\frac{1}{\log {k}_{n}}$	The number of common neighbors with each of them normalized by the logarithm of their degree
Resource Allocation Index (RA)	${\sum }_{{w}_{n}\in {{{\Gamma }}}_{i}\cap {{{\Gamma }}}_{j}}\frac{1}{{k}_{n}}$	The number of common neighbors with each of them normalized by their degree
Local Path Index (LPI)	*S*_*ij*,2_ + *ϵS*_*ij*,3_	The first term represents the number of paths of length equal to 2 between the node *i* and *j*. The second term is the number of paths of length equal to 3 between the node *i* and *j* damped by parameter *ϵ*. We set *ϵ* = 0.01.

To illustrate the differences between the features of retained and removed edges, we use data from the January 2014 snapshot for both transportation networks and present the distributions of those 11 unweighted topological features and the weight for both retained and removed edges. We compared the feature samples of retained and removed edges using the Kolmogorov-Smirnov statistics, a test for the null hypothesis that two samples are drawn from the same continuous distribution. Because we make multiple comparisons, we used Bonferroni corrections on the significance level, i.e. *α* = 0.05/12, where 12 is the number of comparisons. We can reject the hypothesis that the features of retained and removed edges come from the same distribution, with *p* value < 3 × 10^−4^ for all cases.

In order to select a classification model, we performed a stratified 10-fold cross-validation on the balanced training set with 27 widely used classification algorithms available in the *scikit-learn* Python library^35^ and in the *eXtreme Gradient Boost* package^36^. We calculate the balanced accuracy, the F1 score, and the area under the receiver operating characteristic curve (ROC-AUC) to compare the classification performance of the 27 algorithms (see Methods for implementation details and Supplementary Fig. S1 for the performance of all algorithms). For 8 of the 27, we obtain high and stable accuracies (Fig. 2c). The results suggest that those algorithms have consistent and similar prediction accuracies ranging from 0.6 to 0.8. We select a single algorithm with high accuracy and low error variance, XGBClassifier, for all subsequent analyses.

### Prediction

We consider four separate models using unweighted topological features, weighted topological features (Table.S1), edge weights, unweighted topological features & edge weights as the feature vectors for the XGBClassifier.

#### Simultaneous prediction

**Fig. 3:**
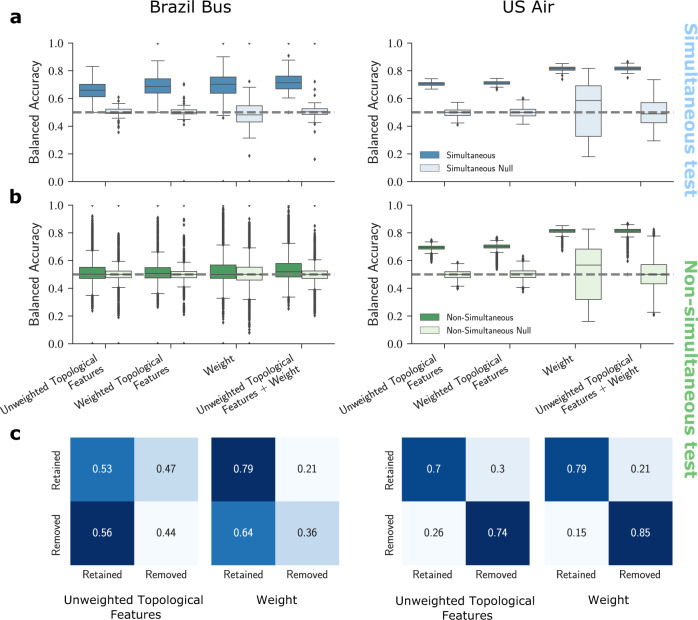
Comparison of different models’ performances against appropriate null models. The box plots show the balanced accuracies for (**a**) simultaneous tests and (**b**) nonsimultaneous tests on all time steps for bus transportation network and air transportation network using unweighted topological features, weighted topological features, edge weights, and unweighted topological features + edge weights. **c** Confusion matrices for the model's non-simultaneous predictions for a snapshot of the Brazil Bus net and a snapshot of the U.S. Air net. Even though the model of the Brazil Bus net is able to perform well on the simultaneous test, its performance on the non-simultaneous test is poor.

Considering only unweighted topological features, for the Brazil Bus net, the balanced accuracies using the XGBClassifier in simultaneous tests have an average of 0.65 (Fig. 3a). For the U.S. Air net, XGBClassifier yields an average balanced accuracy of 0.70. These results suggest that with this ML approach we can differentiate the retained edges from the removed edges in a given network snapshot using their topological features.

Considering weighted topological features marginally increases the balanced accuracy to 0.69 for the Brazil Bus net and 0.71 for the U.S Air net. In contrast, edge weights alone improve the predictive power of the model by 10% to 0.82 for the U.S. Air net. Including both unweighted topological features and edge weights does not significantly improve the models’ performance compared with the model that only uses edge weights to classify removals.

#### Nonsimultaneous prediction

A more general and useful test, however, is achieved by using a model trained on a single snapshot to predict edge removals in latter snapshots. Surprisingly, the prediction of the XGBClassifier for the non-simultaneous tests in the Brazil Bus net is no better than random guessing (Fig. 3b). For the U.S. Air net, the model yields an average balanced accuracy of 0.70 using topological features and 0.82 using edge weights, similar to what was observed for simultaneous prediction. See Fig. 3c for confusion matrices.

It is not surprising that weights are the most predictive feature of the model for the US Air net. Connections with more flights are likely to include services from different airlines, and thus unlikely to suddenly drop to zero. Surprisingly, the same argument does not hold for the Brazil Bus net.

### Model interpretation

**Fig. 4:**
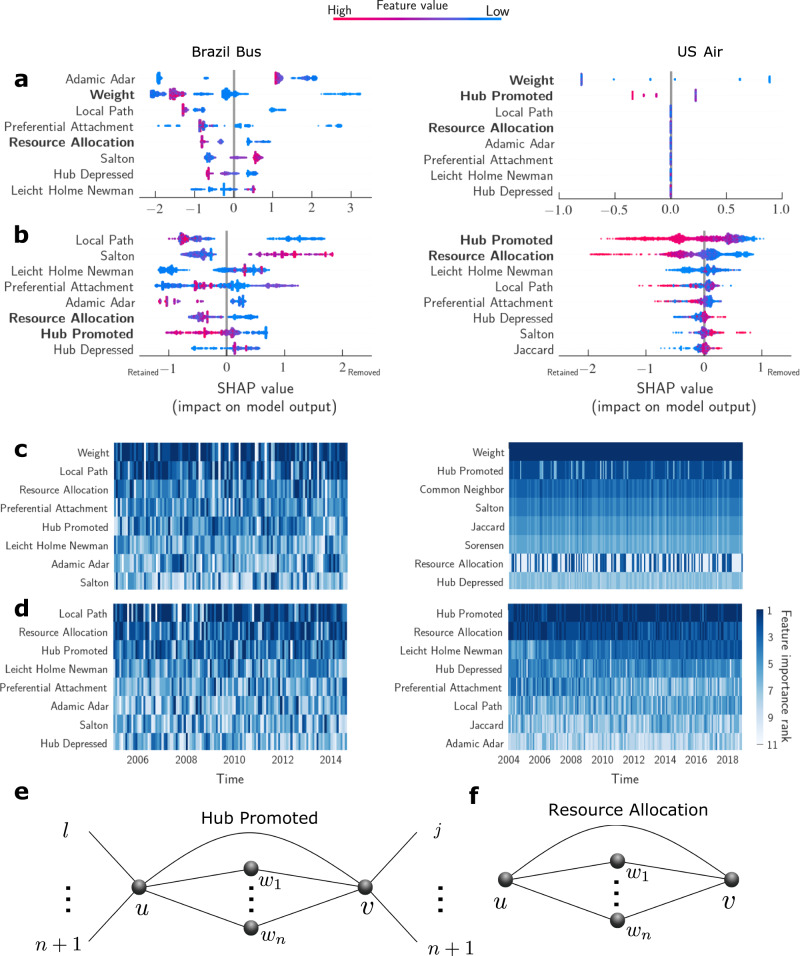
Edge weight, the hub promoted index and the resource allocation index consistently have the largest predictive power for whether edges will be removed or retained across different snapshots for the U.S. Air net. **a** Summary of SHAP values for the simultaneous test of the January 2008 Brazil Bus net and the January 2008 U.S. Air net. We use SHAP values to quantify the importance of features and how they affect the prediction. The feature importance is ranked by the sum of SHAP value magnitudes from top to bottom. For every feature, each point represents a data observation and the color shows its corresponding value. The impact on model output is shown on the x-axis. Positive values correspond to the removed edges, and negative values correspond to the retained edges. In the model with both unweighted topological features and edge weights, Adamic and weight are the most important features in the Brazil Bus net. For the US Air net, the weight is the most dominant feature and the unweighted topological features are less significant. **b** In the model with only unweighted topological features, the most predictive features are the local path index and Salton index for the Brazil Bus net. The hub promoted index and the resource allocation index are the most predictive features for the U.S Air net. It means edges with low values of the hub promoted index and the resource allocation index are more likely to be removed. **c** Ranking of features importance for all snapshots according to their SHAP values. The darker the bar, the more important a feature is for that snapshot. If the ranking changes over time, then a predictive model is accurate only for the time for which it was fitted. Feature rankings are quite stable for the U.S. Air net, but not for the Brazil bus net. Weight is consistently the most important feature for the U.S. Air net. **d** The results are similar when edge weights are not included. **e** Calculating the hub promoted index *h*_*p*_ of the edge connecting *u* to *v*. If *k*_*u*_ < *k*_*v*_, *h*_*p*_ can be seen as the fraction of node *u*'s neighbors that are connected to node *v*. A large fraction suggests node *v* is likely a hub thus a direct connection between *u* and *v* is important. **f** Calculating the resource allocation index *r*_*a*_ of the edge connecting *u* to *v*, *k*_*n*_ is the degree of their common neighbor node *w*_*n*_. Larger *n* means greater redundancy of short paths from *u* to *v*. However, not all redundancy is the same. Redundancy through highly connected nodes (*r*_*a*_ is small) would suggest that a direct connection is not important, but redundancy through poorly connected nodes (*r*_*a*_ is large) would mean *u* and *v* are likely hubs and their connection is important.

Next, we investigate why our predictions fail for the Brazil Bus net in the non-simultaneous tests. To this end, we use the SHapley Additive exPlanations (SHAP) values^10,37,38^. Figure 4a shows the SHAP values summary of the feature importance as well as how their values affect the outputs of the model for the simultaneous test in a particular snapshot considering the model with edge weights and topological features. The SHAP values summary for a particular snapshot reveals that the Adamic-Adar index, edge weights, and the local path index are the most important feature for the bus network. For the U.S. Air net, the most important features for predicting edge removals are edge weights, the hub promoted index and the local path index.

Figure 4b shows the same SHAP values summary for the model considering only the unweighted topological features. For the U.S. Air net, the most important unweighted topological features for predicting edge removals are the hub promoted index and the resource allocation index. Specifically, edges with low values of the hub promoted index and the resource allocation index are more likely to be removed. The ranking of feature importances remains quite stable for different time snapshots.

While edge weight does not unveil the dynamics of the network and is on average ranked the most important feature at all snapshots (Fig. 4c), the hub promoted index and the resource allocation index are consistently the most important features in determining which edges are removed (Fig. 4d) in the U.S. Air net. This implies that the U.S. Air net has consistent removal dynamics over time, whereas, the Brazil Bus net shows no such stability.

For the bus network, features such as the local path index are important in some snapshots but not in others. This variability in the ranking of feature importance explains why non-simultaneous predictions fail for the Brazil Bus net: The model over-fits the current snapshot and performs well on the simultaneous test but fails to generalize when predicting edge removals on different snapshots.

For the U.S. Air net, the primacy of edge weights as the most important feature is not surprising. Edges tend to maintain their weights over time, so edges with low weights are less likely to be retained. However, this is tautological because it does not advance our knowledge about how edge weight is determined. The weight of an edge can be predicted using unweighted topological features (Fig. S3). To this end, we look at the unweighted topological features.

To the predictions of edge removals, the large predictive power of the hub promoted index and the resource allocation index can be understood if one considers that they capture the importance to a city of maintaining connections to hubs^34,39,40^. The hub promoted index is defined as

2 $${h}_{p}=\frac{\left|{{{\Gamma }}}_{u}\cap {{{\Gamma }}}_{v}\right|}{\min ({k}_{u},{k}_{v})}.$$

If *k*_*u*_ < *k*_*v*_, *h*_*p*_ can be seen as the fraction of node *u*’s neighbors that are connected to node *v*. A large value suggests node *v* is likely a hub (compared with non-hubs, node *u* shares more common neighbors with hubs) thus a direct connection between *u* and *v* is important (Fig. 4e). The resource allocation index is defined as

3 $${r}_{a}=\mathop{\sum }\limits_{{w}_{n}\in {{{\Gamma }}}_{u}\cap {{{\Gamma }}}_{v}}\frac{1}{{k}_{n}},$$

where *k*_*n*_ is the degree of the common neighborhood node *w*_*n*_. As Fig. 4f illustrates, larger *k*_*n*_ means greater redundancy of short paths from *u* to *v*. However, not all redundancy is the same. Redundancy through highly connected nodes (*r*_*a*_ is small) would suggest that a direct connection could be replaced by a 2-step path through hubs, but redundancy through poorly connected nodes (*r*_*a*_ is large) would mean *u* and *v* are likely hubs and that their connection is important.

### Model performance during the COVID-19 pandemic period

The U.S. economy was strongly affected by the COVID-19 pandemic. Coupled with travel restrictions, the economic downturn produced a strong reduction in airline traffic. Between February 2020 and April 2020, the number of monthly passengers on US domestic flights collapsed from 70 million to 2.87 million. This extraordinary situation provides us with a natural experiment with which to test the ability of our approach to continue making accurate predictions in the face of external shocks.

**Fig. 5:**
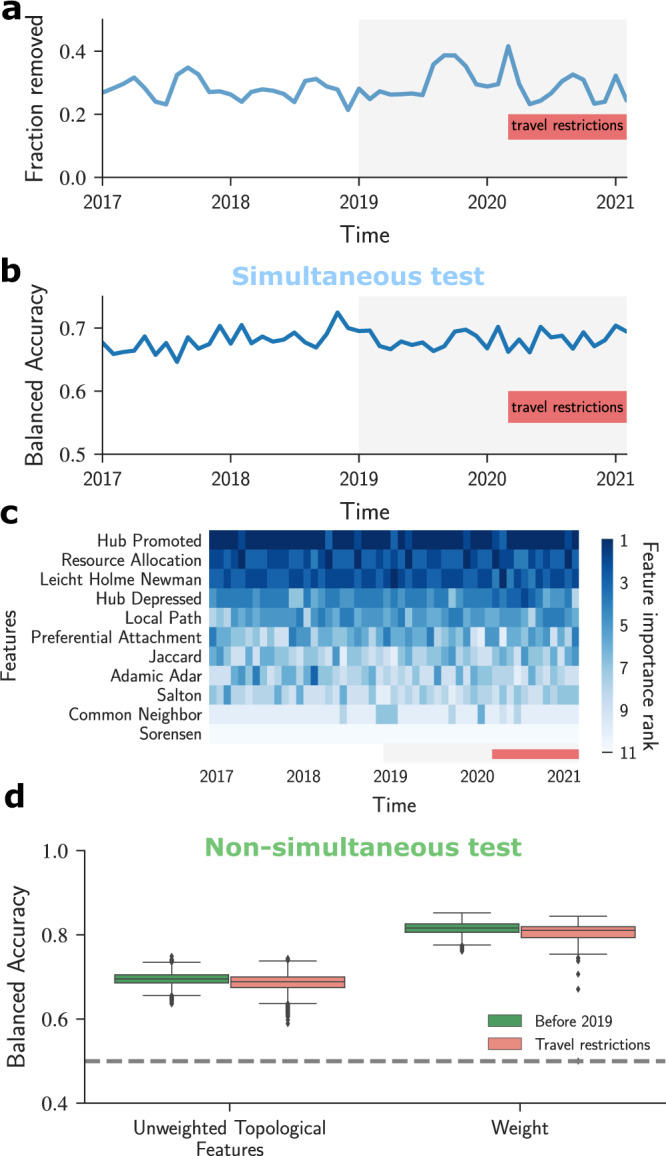
Forecasting edge removals in the U.S. air transportation network during a large external shock. **a** Fraction of edges removed monthly remains similar to the pre-pandemic period, despite the sharp reduction in the number of passengers flying during the COVID-19 pandemic^47^. The grey shaded area highlights the new data obtained for this analysis. The red bar indicates the period under the travel restrictions. **b** The balanced accuracy of simultaneous tests as a function of time shows that the model is able to identify removed edges under the external shock caused by the travel restrictions. **c** Ranking of feature importance according to their SHAP values for each snapshot in the period of travel restrictions. Similar to the pre-pandemic period, the hub promoted index and the resource allocation index remain the most predictive features. **d** Results for the non-simultaneous tests are consistent for both pre- and postpandemic's travel restrictions. The box plot shows the balanced accuracy for the periods before and during the travel restrictions.

We downloaded the data needed to construct the U.S. air transportation network for the period January 2019 to March 2021. We found that despite the sharp reduction in the number of passengers, the fractions of edges removed monthly from the air transportation network were similar to those observed in the pre-pandemic period (Fig. 5a).

To test the accuracy of our model in predicting edge removals during the period of travel restrictions, we first considered the simultaneous test. We obtained very similar balanced accuracy results for this period when compared to the period before the travel restrictions, suggesting that the considerations used to make decisions about which connections to remove remained consistent during the later period (Fig. 5b). We also found that the ranking of feature importance was also consistent after the travel restrictions were in place, making our model suitable for predictions even under this exogenous shock (Fig. 5c). Finally, we investigated our model using the non-simultaneous test to compute the prediction accuracy during the travel restriction period (Fig. 5d). Despite the small reduction in the accuracy for the months after the travel restrictions, the balanced accuracies obtained are very similar.

### Long-term stability of forecast

Using edge weights as a feature in our classification model yields higher accuracy. Whereas predicting whether edges are going to be removed or not suffices to build sensible scenarios for the future of transportation networks, the higher accuracy of the model using edge weights imposes the challenge of predicting the weights of future snapshots. A shortcoming to such a model is that it needs another model to predict edge weights, increasing the overall complexity of the approach. To test the feasibility of such an approach, we take steps in this direction and check the stability of a model that uses only weights as features for long-term predictions. To compute the edge weights in future snapshots, we first fit a regression model that uses the weights of the current snapshot to predict the weights of the next snapshot, this is, *w*_*ij*_(*t*) = *f*(*w*_*ij*_(*t* − 1)). For edges that are added in future snapshots, we input the weights directly from the data so that we do not need to introduce an additional model to predict edge additions.

**Fig. 6:**
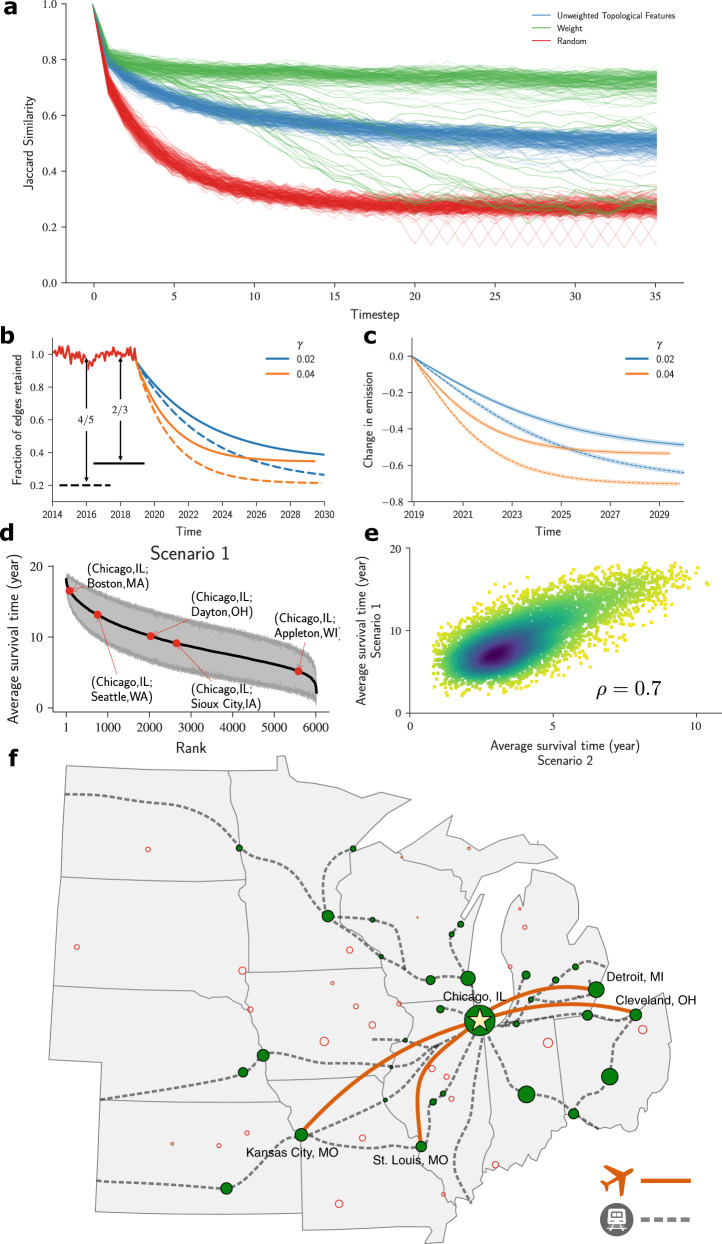
Forecasting the impacts of a hypothetical reduction of the scale of the U.S. Air net. **a** Simulations of long-term forecast for the unweighted topological features model (blue line) edge weights (green line), and random removals (red line). The edge weight model shows better performance but larger variability (green line). The good results of the edge weights model are called into question, because predictions produced by this model can be no better than random removals at the end of the simulation (see red lines). Despite the lower performance of the model that only uses unweighted topological features (blue line), this model is preferable for long-term predictions because predictions are consistent independent of the month chosen to train the model. **b** Starting with the most recent network in our data set (December 2018), we remove edges at a constant rate. Fraction of edges retained in the network for four different scenarios. **c** Estimated change in *CO*_2_ emissions. The lines show the averages of estimations and the shaded areas show the 95% confidence intervals. **d** Susceptibility of an edge to removal for a simple scenario: *R*_*f*_ = 2/3 and *γ* = 0.02. The edges are ranked by their average survival time obtained from 30 simulations. The grey envelope shows the 95% confidence intervals. Note that hub-to-hub connections like (Chicago, IL - Boston, MA) have a greater survival time. **e** Comparison for two scenarios: *R*_*f*_ = 2/3 and *γ* = 0.02 (scenario 1) v.s. *R*_*f*_ = 4/5 and *γ* = 0.04 (scenario 2). We find a Pearson's correlation coefficient of 0.7 between the average survival time in the two scenarios. While this correlation is quite high, it nonetheless means that actual survival time cannot be a priori predicted perfectly. **f** A possible scenario for air and rail transportation in 2035. Air connections are predicted using scenario 1 from above. Rail connections are from the “Amtrak 2035” plan and are represented by the dashed gray lines. Air connections are represented by the orange lines. Red hollow circles represent cities that would be left without air or rail connections. Green circles represent cities that would be connected by rail. Only four cities would have both air and rail connections. Note that since none of the rail connections are planned to be high-speed, rail travel would not be competitive against automobile travel. The map was generated using the Python package *cartopy* version 0.20.0.

We simulate long-term forecast for the U.S. Air net for a model considering only unweighted topological features and a model considering only edge weights (Fig. 6a). Starting from a given snapshot network *G*_*m*_, the model is trained to predict the edges removed in the next snapshot *G*_*m*+1_. Then, the model uses the prediction *G*_*m*+1_ to predict *G*_*m*+2_ and so on and so forth. To evaluate the performance of the predictions at each time step, we compare the structure of the predicted network with the structure of the actual network using the Jaccard similarity

4 $$J=\frac{\left|{E}_{data}\cap {E}_{predicted}\right|}{\left|{E}_{data}\cup {E}_{predicted}\right|}$$

where *E*_*data*_ is the edge set from the actual network and *E*_*predicted*_ is the edge set of the predicted network.

Our analysis suggests that unweighted topological features have more stable predictions independently of the initial conditions of the network, that is, which month was chosen to train the model. In contrast, models considering only the edge weights yield about 13% of trajectories with very large errors, suggesting that this model could become unexpectedly poor for long-term predictions. In fact, at the end of the simulation, models using only edge weights can, at times, have a performance similar to a model where edges are removed randomly from one month to another (Fig. 6a).

### Forecasting changes to the U.S. Air net

Encouraged by the ability of our approach to forecast the edge removal dynamics of the U.S. Air net over long periods, we next use the model considering unweighted topological features to simulate the effect of hypothetical air travel restrictions aiming to reduce *CO*_2_ emissions. We use the model trained on a known snapshot to predict the probability that a given edge is removed and remove it according to that probability. We take the December 2018 snapshot of the U.S. Air net as the initial state of the network. In each simulation, we assume that there is a target *N*_*f*_ for the total number of edges in the network and that at each time step we remove a fraction of existing edges

5 $$\delta {N}_{m}=-\gamma ({N}_{m}-{N}_{f}),$$

where *m* is the number of months from the start of the simulation and *N*_*m*_ is the number of edges in the current snapshot.

Figure 6 b shows ensembles, each including 30 simulations starting on December 2018 and removing (*R*_*f*_ = 2/3, 4/5, where *R*_*f*_ = 1 − *N*_*f*_/*N*_0_) of edges at two different rates (*γ* = 0.02, 0.04). Based on the predicted edge removals, we project the estimated carbon emissions relative to the emissions in 2018 (see Fig. 6c and the SI for details of the estimations)^41^. To better quantify the likelihood of removal, we calculate the average time an edge is retained and rank edges from the shortest survival time to the longest survival time (Fig. 6d). We find that edges connecting hubs (e.g. Chicago, IL, and Boston, MA) are the least likely to be removed. Of practical importance, we find that an edge’s survival time depends on the values of the two parameters, *γ* and *R*_*f*_ (Fig. 6e).

Daily global *CO*_2_ emissions decreased by 17% during the early stages of the COVID-19 pandemic. A reduction in carbon emissions this dramatic could help many countries achieve the goals of the Paris Climate Agreement^10^. An important component of such reduction would be decreasing the number of air connections among cities such as the one we explored above. An important question, thus, is the extent to which our model may be of use to planners.

As Fig. 6f makes clear, many Midwestern cities are likely to lose air connections if there is a large reduction in the size of the U.S. domestic air transportation network. Only a fraction of those cities have or will host new rail connections. For the most part, the existing or proposed rail connections will not be served by high-speed trains. In practical terms, this means that most travelers between those cities will do so by automobile.

The removal of a large fraction of air connections could thus lead to an increase in automotive traffic for trips in the 3-8 hours range. Such an increase could result in increased *CO*_2_ emissions due to the construction, expansion, and maintenance of roads and due to increased miles traveled by car. This might be a missed opportunity. High-speed rail is a scalable, and a more environmentally sound approach to transportation than adding cars to the roads, and building and expanding roadways.

A complicating factor is the time scale for the planning of new U.S. rail lines. Amtrak has advertised its plans under the name “2035 Amtrak”^42^. Much can and will change in the U.S. between now and 2035. For instance, climate change is likely to force migrations from the South and West of the U.S. areas of the Midwest. Thus, our model cannot provide the answer to how to plan a rail system that will anticipate population and air transportation changes. However, our model can help planners build additional scenarios for future changes in the U.S. domestic air transportation network.

## Discussion

We show that edge removal processes in transportation networks are not random and that it is possible to make accurate predictions based on local network structures. Even though those features are able to differentiate edges removed and retained, a model trained in a single time snapshot is not able to correctly predict removed edges in different time snapshots for the Brazil Bus net. In contrast, for the U.S. Air net, the non-simultaneous tests show that the features of the edges removed are similar in all snapshots.

For the U.S. Air net, we find that edge weight, the hub promoted index, and the resource allocation index consistently have the largest predictive power for the aggregate network. This finding is not surprising since it simply highlights the fact that direct connections between hubs are very important while connections to a city already connected to a hub are not. Remarkable, when considering individual airlines, we find that the hub promoted index is still very important for accurate prediction, but its importance varies over time and by the airline. That is, predicting the removed edges for the aggregate network is actually simpler than the prediction for an individual airline (see Supplementary Fig. S4 and Fig. S5).

Our study has several limitations. First, the number of topological features tested in our work is limited. The predictive power could be improved by including additional features. However, we did test the impact of some global features such as the edge betweenness centrality, edge current flow betweenness centrality, and demographic features such as the intercity gravitation flow^43^, but did not see any improvement in predictive power (see Supplementary Fig. S6 and Fig. S7). We also compared our model to a purely physical model that removes edges according to the rank of inter-city gravitation flow (see Supplementary Fig. S8). Our analyses thus suggest that the local features can predict edge removals better than global and demographic factors.

Second, we left the interplay of the multilayer structure of the air transportation networks unexplored^44^. For example, it would be very interesting to use machine learning approaches to explore the interplay between the removal of edges in one network (e.g., air transportation network) and the growth of edges in another transportation network (e.g., high-speed rail systems).

Third, the crucial role of airline strategy and route network optimization is not explicitly included in our approach. Nonetheless, we note that many events concerning airline strategy that is arguably impossible to predict – governmental regulation, airport expansions, mergers and acquisitions, bankruptcy protection, epidemics, natural disasters, and so on – would also affect the decision to drop routes. It is thus remarkable that our approach is able to achieve such accuracy when predicting changes in the aggregate network.

Finally, it is widely accepted that exogenous shocks to the economy or from the environment can dramatically change the topology of the network as airline companies try to adapt to the new market conditions. Surprisingly, the fact that local topological features alone can enable accurate predictions about edge removals in the air transportation network highlights the importance of our results to the literature on transportation networks. Nonetheless, an interesting question for further research would be the investigation of possible market scenarios where removals of airline connections are not only a function of topological features but also of other external factors such as large-scale migration patterns due to climate change.

## Methods

### Data

The Brazilian inter-cities bus data was collected from the Brazilian National Land Transportation Agency (ANTT)^31^. The dataset contains all inter-cities bus transportation from January 2005 to December 2014 with monthly resolution. The data is represented as a temporal unweighted undirected network, where nodes are individual bus stops (cities) and edges represent bus routes between the two cities within that monthly snapshot. The network has 120 snapshots with about 1734 nodes and 18781 edges on average.

We obtained United States domestic air transportation data from the Bureau of Transportation Statistics (BTS)^33^. The data is in the period from January 2004 to December 2018. We later obtained data for the period from January 2019 to March 2021 for the analysis of the impact of the COVID-19 pandemic’s travel restrictions. Using the same approach we used in Brazil inter-cities bus data, each snapshot of the network is constructed from data of the corresponding month. The nodes are airports (cities) and an edge represents that there is at least one airline connecting the two cities within that monthly snapshot. The networks have 192 snapshots with about 819 nodes and 6547 edges on average.

### Class balancing

Most machine learning classification algorithms favor the majority class in an imbalanced dataset. In the two transportation networks we study, the number of removed edges is much smaller than the edges that are retained. To mitigate this issue in our highly imbalanced data, we balanced the training data by keeping the same number of the majority class data samples (retained edges) as the minority class data samples (removed edges) using random under-sampling.

### Performance metrics

The performance metrics are calculated using *Scikit-learn* (version: ‘0.21.3’) built-in functions^35^. For binary classifications, the results fall into true positive/TP (removed edges predicted to be removed), false positive/FN (retained edges predicted to be removed), true negative/TN (retained edges predicted to be retained), and false negative/FN (removed predicted to be retained).

For a binary case, precision is defined as:

6 $${\mathtt{precision}}=\frac{TP}{TP+FP}$$

and recall is defined as:

7 $${\mathtt{recall}}=\frac{TP}{TP+FN}$$

The balanced accuracy is defined as the arithmetic mean of true positive rate and true negative rate:

8 $${\mathtt{balanced-accuracy}}=\frac{1}{2}\left({\mathtt{recall}}+\frac{TN}{TN+FP}\right)$$

The F1 score is defined as the harmonic mean of precision and recall:

9 $$F1=2\times \frac{({\mathtt{precision}}* {\mathtt{recall}})}{({\mathtt{precision}}+{\mathtt{recall}})},$$

The area under the receiver operating characteristic curve (AUC-ROC) is a performance measurement for the classification problems at various threshold settings. It measures the area under the curve of the plot of true positive rates vs. false positive rates. The higher the AUC-ROC, the better the model is in predicting the correct classes.

### Hyperparameter tuning

For XGBClassifier, we performed a brute force grid search hyperparameter tuning. For the sake of computational time, we tested on a predefined hyperparameter space. That is learning rate 0.01–0.4; gamma 0.0–0.2; maximum tree depth 0–10; number of boosting rounds 0–200. All default hyperparameters are outside the region of overfitting and underfitting (see Supplementary Fig. S2). For all analyses, we report our results using the default hyperparameters.

Those hyperparameters are: learning rate = 0.3; number of boosting rounds = 100; maximum tree depth = 3; objective = binary:logistic; booster = gbtree; gamma = 0; min child Weight = 1; max delta step = 0; subsample = 1; colsample bytree = 1; colsample bylevel = 1; colsample bynode = 1; reg alpha = 0; reg lambda = 1; scale pos Weight = 1; base Score = 0.5.

### Null model

To justify that the predictability comes from the non-trivial section of removed edges, we construct a null model to estimate the fraction of correct predictions that XGBClassifier would make if edge features were not correlated with removals. To do so, we shuffle the labels (retained and removed), destroying any correlation between features and labels. Then, we split the data as we did for the original model and train the XGBClassifier algorithm on the training set and test the predictions on the testing set.

If features are not correlated with labels and the null model produces predictions similar to the original model on the non-shuffled data, the predictions observed in non-shuffled data is likely a result of overfitting. If the null model produces predictions that are no better than chance, our ML approach is capturing the functional relationship between edge features and edge removals on the non-shuffled data.

### SHAP (SHapley Additive exPlanation) values

The computation of SHAP values is a suitable approach to quantify feature importance^45^. To assess the importance of a feature, one calculates the change in the expected model prediction by withholding that feature. Mathematically, this method retrains the model on all subset of features *S* ⊂ *F*, where *F* is the set of all features. Since multiple subsets satisfied $S\subseteq F\setminus \left\{i\right\}$, the importance of the feature is computed using all possible permutations. Mathematically, the SHAP value for a particular feature *i* (out of *F* total features), given a prediction *x* is:

10 $${\phi }_{i}(x)=\mathop{\sum}\limits_{S\subseteq F\setminus \left\{i\right\}}\frac{\left|S\right|!\left(\left|F\right|-\left|S\right|-1\right)!}{\left|F\right|!}\left[{f}_{S\cup \left\{i\right\}}\left({x}_{S\cup \left\{i\right\}}\right)-{f}_{S}\left({x}_{S}\right)\right]$$

where ${f}_{S\cup \left\{i\right\}}$ is a model trained with feature $S\cup \left\{i\right\}$, and *f*_*S*_ is a model trained on *S* without feature *i*. Thus, the rank of feature importances are given by the sum of the SHAP value magnitudes *ϕ*_*i*_ over all predictions.

### Estimate the *CO*_2_ emission reduction

To estimate the *CO*_2_ emissions from the U.S. domestic air transportation, we use the average fuel efficiency of U.S. airlines in 2018. The methodology to calculate the *CO*_2_ emission associated with a specific route can be done as follows^46^:

Monthly *CO*_2_ emissions (in tons) = 3.16 × 32.5 (gram fuel per km) × trip distance (in km) × number of flights each month × 10^−6^ (tons per gram).

Where 3.16 is the constant representing the number of tonnes of *CO*_2_ produced by burning a tonne of aviation fuel. 32.5 g fuel per km was the average fuel efficiency of U.S. airlines in 2018^41^.

## Supplementary information

Supplementary Information

## Data Availability

The Brazilian bus transportation network and U.S. air transportation network are freely available for download at http://antt.gov.br/ and https://www.transtats.bts.gov/TableInfo.asp, respectively.

## Code availability

Our code is available for download at https://github.com/amarallab/transportation_network_evolution.
