# Supplementary material for: Forecasting the evolution of fast-changing transportation networks using machine learning
Source: Nat Commun. 2022 Jul 22;13:4252. doi: 10.1038/s41467-022-31911-2 (PMC9307821; doi:10.1038/s41467-022-31911-2)
Supplement: Supplementary file 1 — Supplementary Information [file 41467_2022_31911_MOESM1_ESM.pdf]

Forecasting the evolution of fast-changing transportation networks using machine learning

Weihua Lei,[1] Luiz G. A. Alves,[2] and Luís A. Nunes Amaral[1,2,3,*]

[1]*Department of Physics and Astronomy,*
Northwestern University, Evanston, IL 60208, U.S.A

[2]*Department of Chemical and Biological Engineering,*
Northwestern University, Evanston, IL 60208, U.S.A

[3]*Northwestern Institute on Complex Systems (NICO),*
Northwestern University, Evanston, IL 60208, U.S.A

[*] amaral@northwestern.edu

SUPPLEMENTARY INFORMATION

Performance of widely used classification algorithms

We performed a stratified 10-fold cross-validation on the balanced training set with 27 widely used classification algorithms. These algorithms are available in the *scikit-learn* Python library [1] and in the *eXtreme Gradient Boost* package [2]. We calculate the balanced accuracy, F1 score, and ROC-AUC to compare classification performance (Fig. S1).

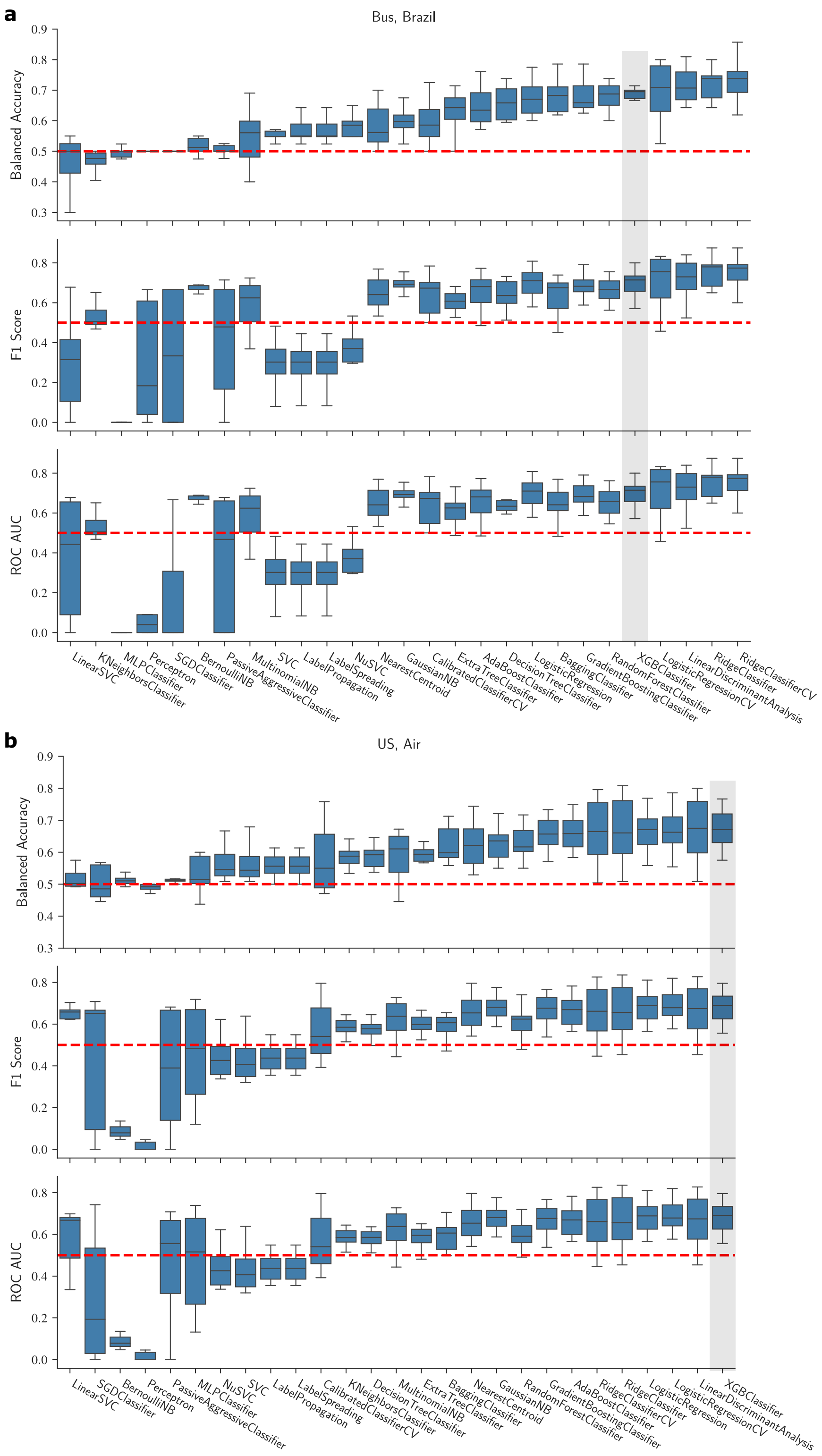

FIG. S1. **Performance of 27 widely used classification algorithms.** We evaluate the performance of 27 common supervised classification algorithms using ten-fold cross-validation. We show box plots of the estimated model balanced accuracies for **(a)** the Brazil Bus net and **(b)** the U.S. air net.

Cross-validation accuracy for grid search

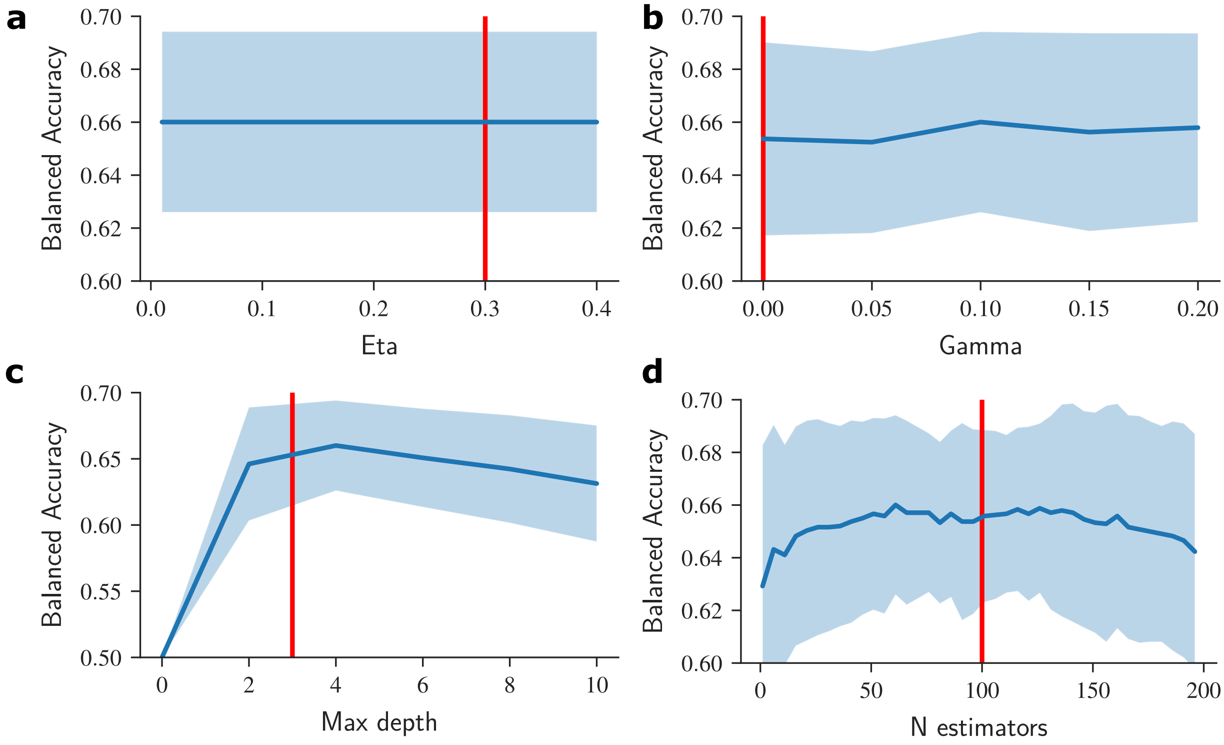

FIG. S2. **Hyperparameter tuning.** Cross-validation accuracy for the grid search using the XGBClassifier algorithm on four important parameters: **(a)** the learning rate (Eta), **(b)** the minimum loss reduction (Gamma), **(c)** the max tree depth (Max depth) and **(d)** number of trees (N estimator). The x-axis shows the searched parameter range and the y-axis shows the balanced accuracy.

Prediction weights on the U.S. air network

To Further extend the applicability of our approach, we generalize our approach to regression tasks of predicting edge weights in the US air net where weights are the number of flights during the observation window (Fig. S8). We found unweighted topological features used for identifying removal edges are able to provide good estimations on the number of flights in both simultaneous and non-simultaneous tests. Despite the good estimations, the number of flights are nosier and more sensitive to societal factors and political policies (e.g. the travel restriction during the pandemics).

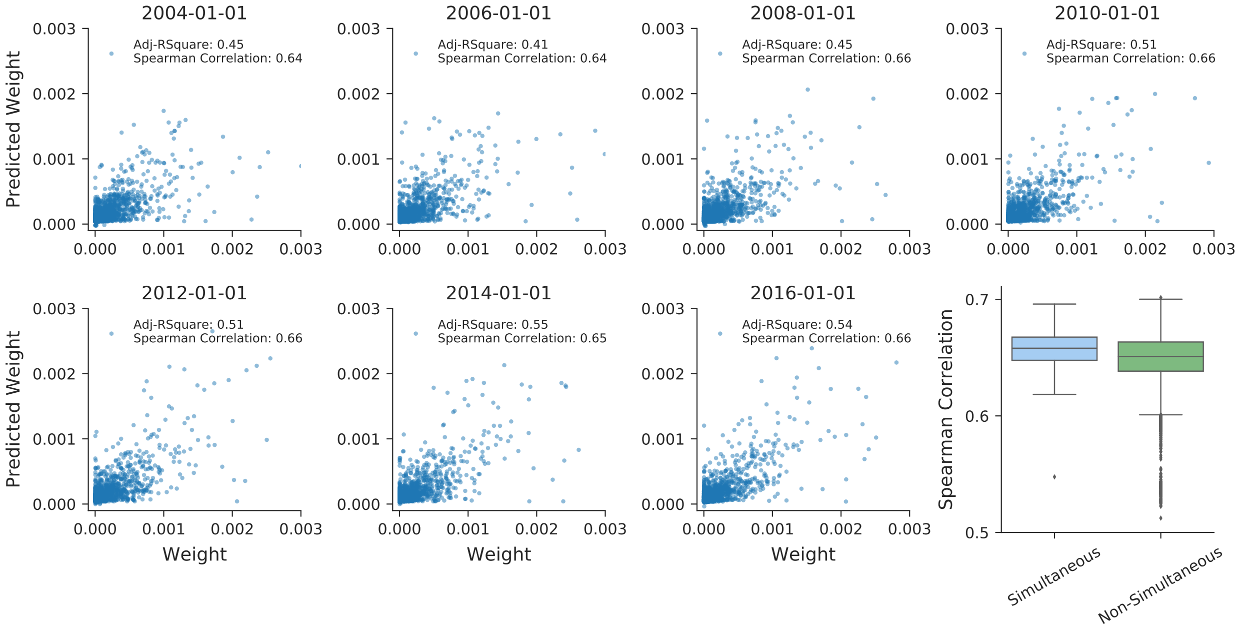

FIG. S3. **Unweighted topological features provide good estimations on the number of flights** The plots show the regression results of models using unweighted topological features to the predictions of the number of flights in U.S. air net during the monthly observation window. Predicted weights (normalized by the total weights) from unweighted topological features are compared with the number of flights (normalized by the total number of flights) for several snapshots of U.S. air net. The last panel shows the summary of Spearman correlations between predicted weights and the number of flights for all available snapshots in the U.S. air net. An average Spearman correlation of 0.65 is achieved for simultaneous tests and non-simultaneous test.

Weighted topological features

TABLE S1. The weighted topological features are calculated by mapping the weighted network into a multigraph [3]. Γ_i refers to the set of neighbors of node i. W is the weighted adjacency matrix. w_i is the weighted degree of node i. w_{ij} is the weight of edge e_{ij}. $\epsilon = 0.01$.

**Feature**	**Definition**
Weighted Common Neighbors (WCN)	$\sum_{w_n \in \Gamma_i \cap \Gamma_j} min(w_{in}, w_{jn})$
Weighted Salton Index (WSA)	$\frac{WCN}{\sqrt{w_i \times w_j}}$
Weighted Jaccard Index (WJA)	$\frac{WCN}{w_i + w_j - WCN}$
Weighted Sørensen Index (WSO)	$\frac{2 \cdot WCN}{w_i + w_j}$
Weighted Hub Promoted Index (WHPI)	$\frac{WCN}{min(w_i, w_j)}$
Weighted Hub Depressed Index (WHDI)	$\frac{WCN}{max(w_i, w_j)}$
Weighted Leicht-Holme-Newman Index (WLHNI)	$\frac{WCN}{w_i \times w_j}$
Weighted Preferential Attachment Index (WPA)	$w_i \times w_j$
Weighted Adamic-Adar Index (WAA)	$\sum_{w_n \in \Gamma_i \cap \Gamma_j} \frac{1}{\log w_n}$
Weighted Resource Allocation Index (WRA)	$\sum_{w_n \in \Gamma_i \cap \Gamma_j} \frac{1}{w_n}$
Weighted Local Path Index (WLPI)	$W_{ij}^2 + \epsilon W_{ij}^3$

Predicting removals at the level of individual airlines

Our data-driven approach to the problem of predicting the evolution of transportation networks does not factor in the role played by individual airlines. The rationale for this choice is our hypothesis that the prediction of route changes for individual airlines would be more challenging than the prediction for the aggregate network. As we show below, our hypothesis is in fact correct. Nonetheless, there are important insights that can be gained from using XGBClassifier with unweighted topological features at the level of individual airlines.

Figure S4 demonstrates that our approach is still able to predict edge removals with an accuracy significantly greater than chance. However, there is a significant decrease in performance when compared to the case for the aggregate network and there is also greater variability in the performance level from year to year. This variability in performance is due to the change in the importance of features over time observed for nearly all airlines (left column of Fig. S4), which contrasts with the consistency of the hub promoted index and the resource allocation index as the two most informative unweighted topological features for the aggregated network.

Interestingly, the variability in features importances may be qualitatively interpreted in the context of the changes experienced by the individual airlines. Southwest Airlines displays the greatest consistency in its features importances. Hub promoted is almost always the most important feature even though during the period considered Southwest Airlines acquired AirTran Airways. In contrast, Delta shows two distinct periods with different features as the most important. Up to 2011, hub promoted is the most important feature, being replaced after that year by the Leicht-Holme-Newman index. This transition aligns well with the time of the merger of Delta and Northwest Airlines which was announced in 2008 and took a few years to finalize.

The degree of temporal change in the most important features is even more dramatic for American Airlines displays. For example, while hub promoted is very important much of the time, during the period 2013–2015, the preferential attachment index becomes the most important feature. This overlaps with the period over which the merger with US Airways was occurring and may be related to route consolidation during the merger. Interestingly, a switch from hub promoted to preferential attachment index occurs again in 2017, per-

haps connected with a decrease in profitability from the record-breaking profitability of the previous 4 years.

Route selection is a matter of great strategic importance to airlines, so one would expect that such critical decisions as which routes to abandon are the result of significant consideration. It is thus quite remarkable that simple heuristics such as the preferential attachment or the hub promoted indices are able to provide significant insight into the outcomes of such decision-making processes. This may be related to the recent discovery by Ahmadi and Zhang that the problem of deciding whether a quadratic function has a local minimizer over an (unbounded) polyhedron, and that of deciding if a quartic polynomial has a local minimizer is NP-hard [4]. Applied to air transportation, this result would imply that airlines cannot really optimize their route networks, and that they are in the end resorting to heuristics.

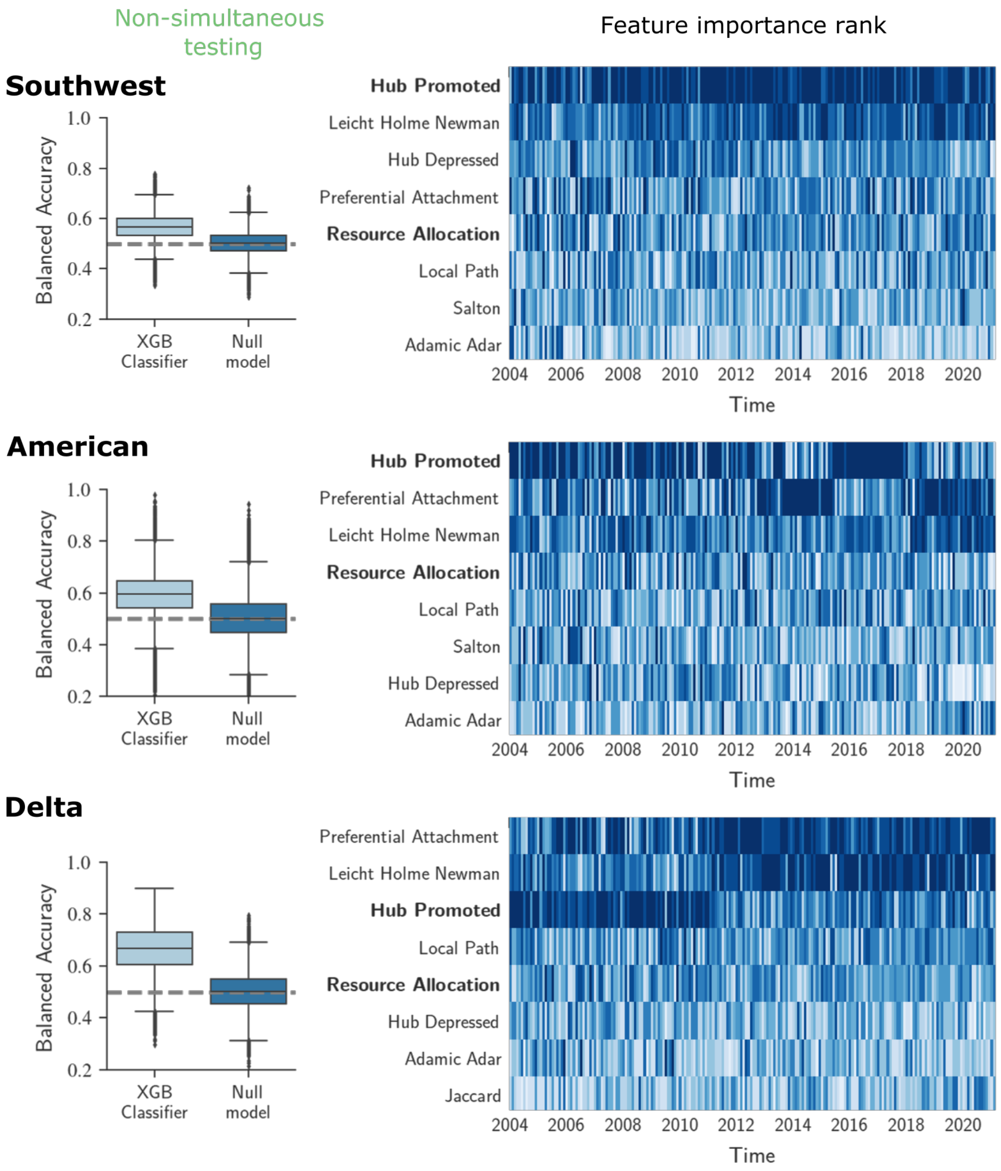

FIG. S4. **Prediction results and removal mechanisms at the level of individual airlines.** We applied our classification approach with unweighted topological features to the route networks of many major airline companies (see SI Fig. S5 for airlines not shown here). Non-simultaneous prediction results and the ranking of feature importance for Southwest Airlines, American Airlines, and Delta Air Line. Our analysis shows that we are still able to predict edge removals with an accuracy significantly greater than chance. Compared to our predictions for the US air net, our models are less accurate, especially for small companies with networks that only cover a small region, and rankings of the feature importance are less consistent.

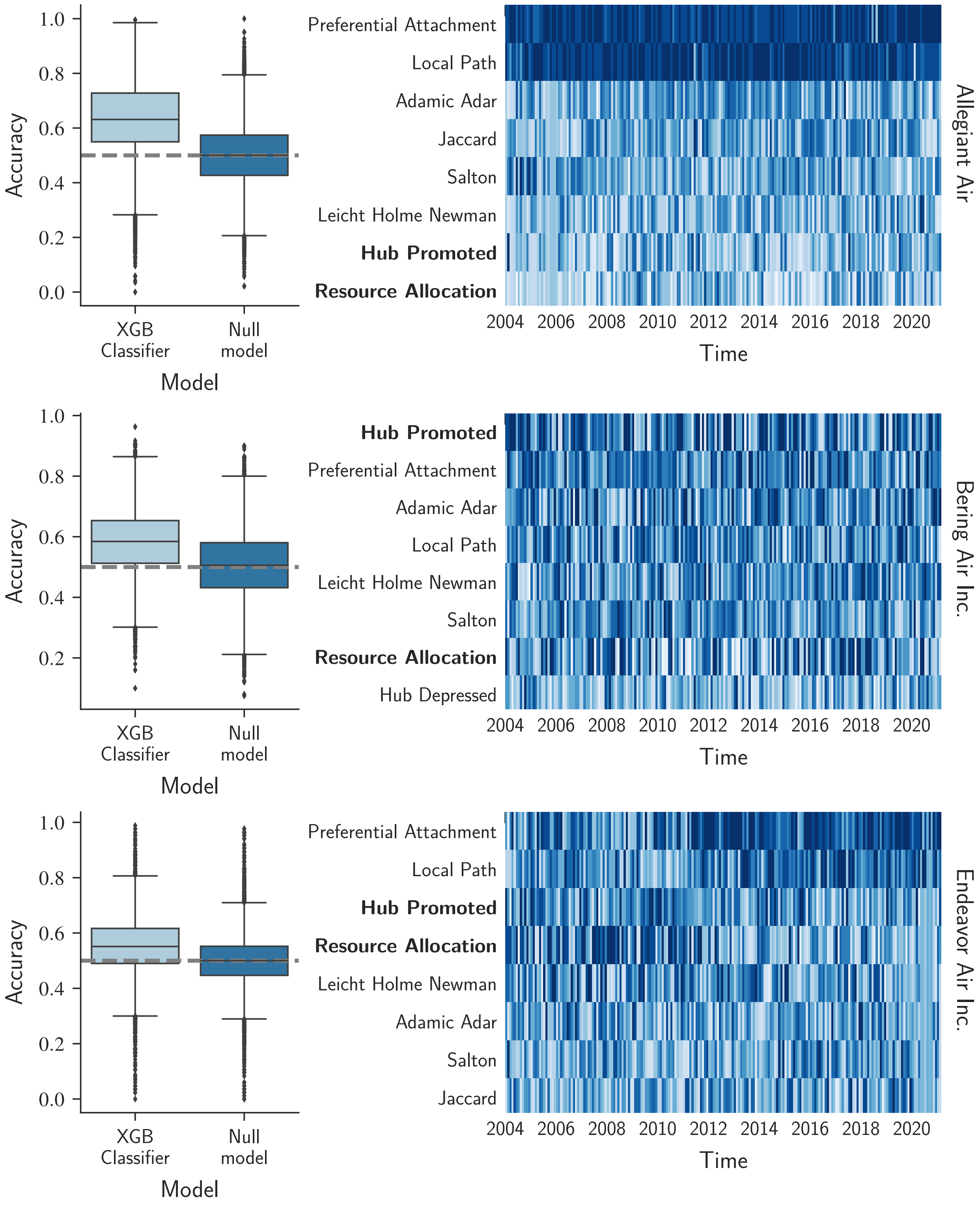

1.0
0.8
0.6
0.4
0.2
0.0
Accuracy
XGB Classifier
Null model
Model
Preferential Attachment
Local Path
Adamic Adar
Jaccard
Salton
Leicht Holme Newman
Hub Promoted
Resource Allocation
2004 2006 2008 2010 2012 2014 2016 2018 2020
Time
Allegiant Air
Hub Promoted
Preferential Attachment
Adamic Adar
Local Path
Leicht Holme Newman
Salton
Resource Allocation
Hub Depressed
Bering Air Inc.
Preferential Attachment
Local Path
Hub Promoted
Resource Allocation
Leicht Holme Newman
Adamic Adar
Salton
Jaccard
Endeavor Air Inc.

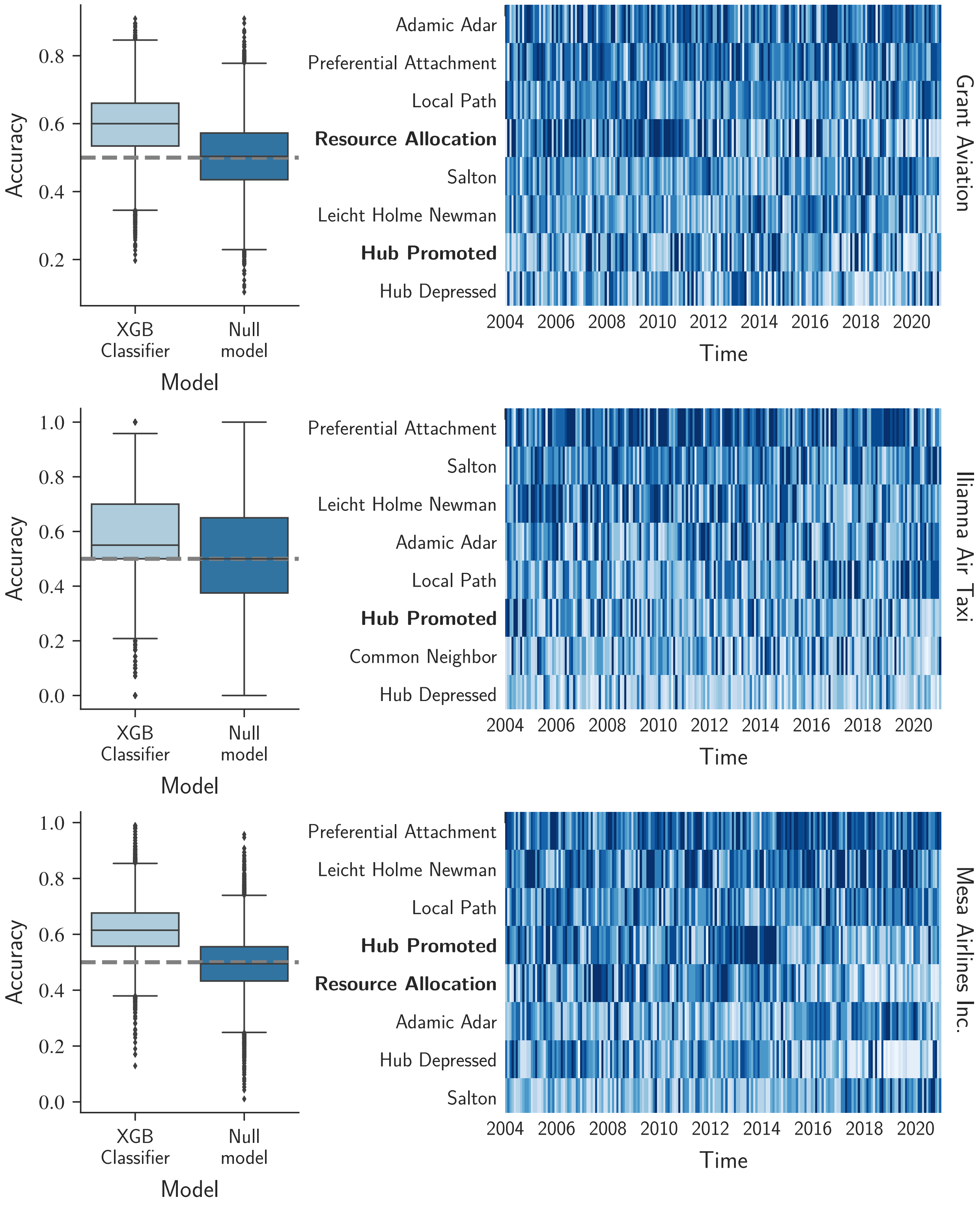

Accuracy
0.2
0.4
0.6
0.8
XGB Classifier
Null model
Model
Adamic Adar
Preferential Attachment
Local Path
Resource Allocation
Salton
Leicht Holme Newman
Hub Promoted
Hub Depressed
2004 2006 2008 2010 2012 2014 2016 2018 2020
Time
Grant Aviation
Accuracy
0.0
0.2
0.4
0.6
0.8
1.0
XGB Classifier
Null model
Model
Preferential Attachment
Salton
Leicht Holme Newman
Adamic Adar
Local Path
Hub Promoted
Common Neighbor
Hub Depressed
2004 2006 2008 2010 2012 2014 2016 2018 2020
Time
Iliamna Air Taxi
Accuracy
0.0
0.2
0.4
0.6
0.8
1.0
XGB Classifier
Null model
Model
Preferential Attachment
Leicht Holme Newman
Local Path
Hub Promoted
Resource Allocation
Adamic Adar
Hub Depressed
Salton
2004 2006 2008 2010 2012 2014 2016 2018 2020
Time
Mesa Airlines Inc.

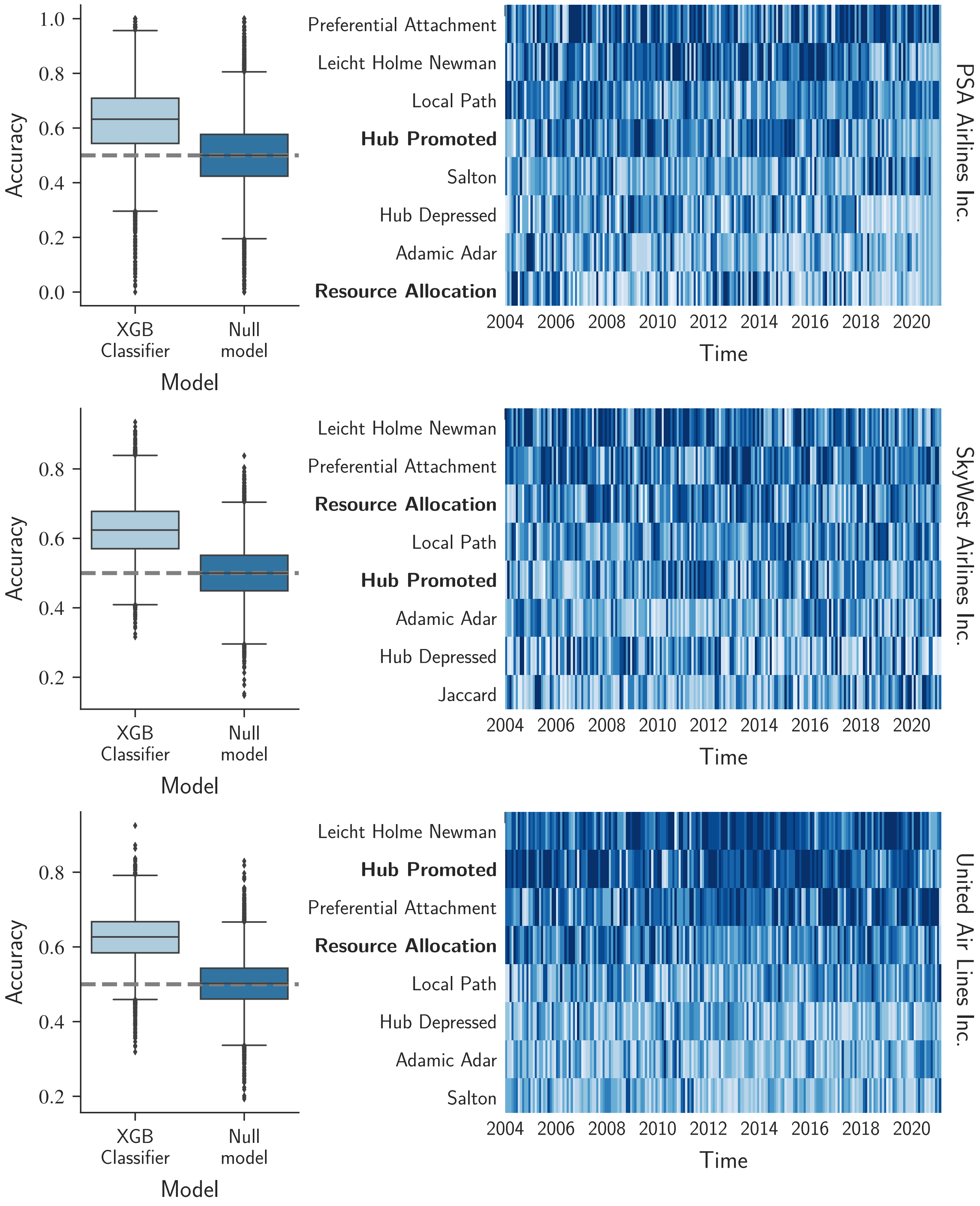

1.0
0.8
0.6
0.4
0.2
0.0
Accuracy
XGB Classifier
Null model
Model
Preferential Attachment
Leicht Holme Newman
Local Path
Hub Promoted
Salton
Hub Depressed
Adamic Adar
Resource Allocation
2004 2006 2008 2010 2012 2014 2016 2018 2020
Time
PSA Airlines Inc.
Leicht Holme Newman
Preferential Attachment
Resource Allocation
Local Path
Hub Promoted
Adamic Adar
Hub Depressed
Jaccard
SkyWest Airlines Inc.
Leicht Holme Newman
Hub Promoted
Preferential Attachment
Resource Allocation
Local Path
Hub Depressed
Adamic Adar
Salton
United Air Lines Inc.

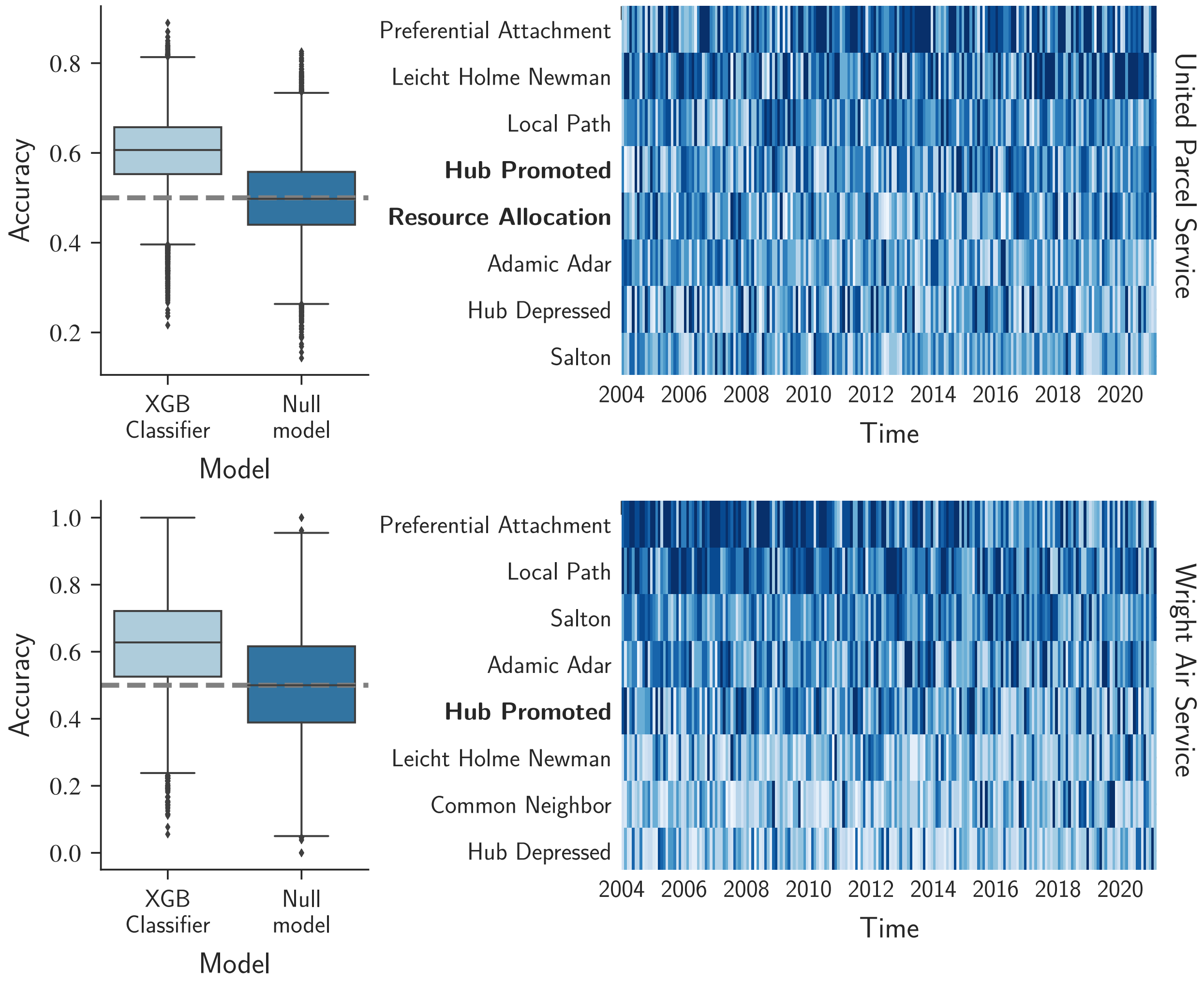

FIG. S5. **Predictions for individual airlines.** We applied our approach to the route networks of all major airlines that are operating during the time window of collected data. The accuracies of non-simultaneous tests and the ranks of feature importance are shown.

Prediction with additional global features

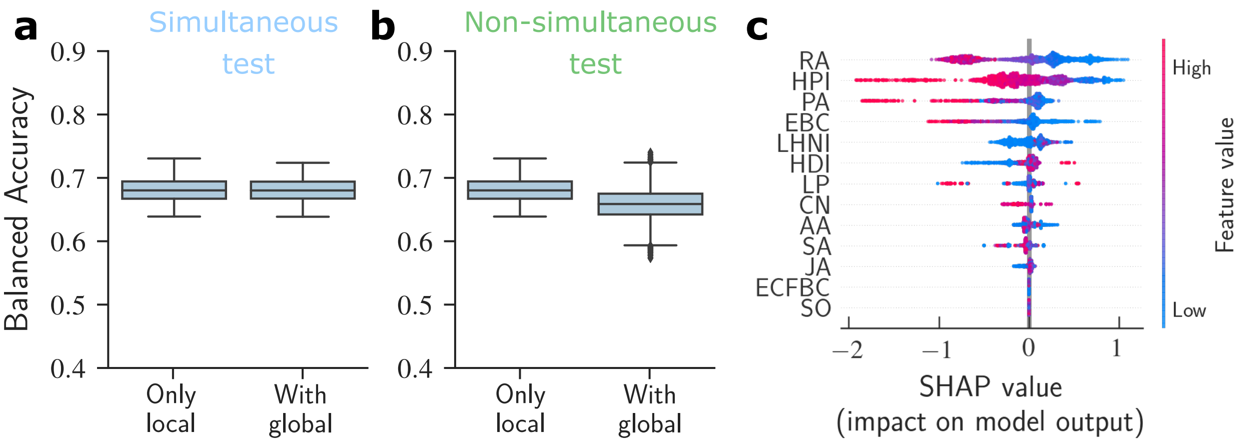

FIG. S6. **Prediction results for models with additional global features.** The box plots show the comparison of model with and without global features. Balanced accuracies for **(a)** simultaneous and **(b)** non-simultaneous tests. The model performance do not improve with additional global features such as the edge betweenness centrality (EBC) and the edge current flow betweenness centrality (ECFBC). **(c)** The SHAP summary plot of the model with additional global features. Local features such as the hub promoted index (HPI) and the resource allocation (RA) are more important than the additional global features in predicting removals.

70 **Predictions with additional demographic gravitation flow as a feature**

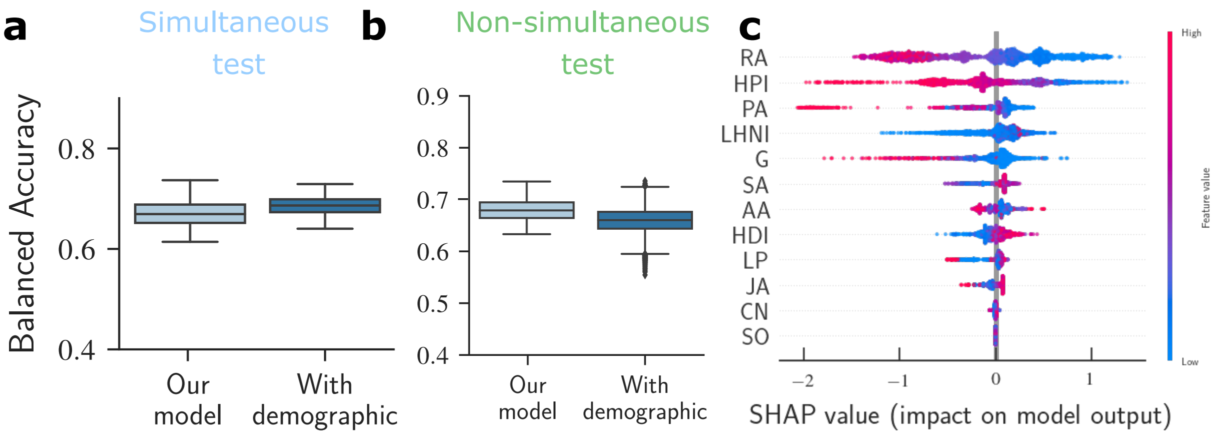

FIG. S7. **Prediction results for including the demographic gravitation flow as a feature.** The box plots show the comparison of the model with only local features and with the model with additional demographic gravitation flow as a feature. Balanced accuracies for **(a)** simultaneous and **(b)** non-simultaneous tests. The model performance does not improve with the additional demographic flow feature. **(c)** The SHAP value summary plot of the model with additional global features. Local features such as the resource allocation (RA) and the hub promoted index (HPI) are more important than the demographic gravitation flow (G) in predicting removals.

Comparison with edge removals predicted by the demographic gravitation flow

In addition to the comparison with the random null model from Fig. **??**, we also computed a model where the edges are removed according to inter-city flow predicted by demographic gravitation "law" ($F_{ij} = N_i N_j / r^2$) [5]. Here, N_i, N_j are the populations of two connected cities obtained from the U.S. 2010 census [6], and r is the distance (in miles) between them. A plausible hypothesis is that connections with high predicted inter-city flows would have larger survival times. This is indeed what we observe S8a. To do so, for each snapshot m, we computed and ranked the edges by the demographic gravitation "law" F_{ij}, and removed N edges with the smallest flow, where N is equal to the number of edges removed in the real data. We show a confusion matrix for the model predictions of one snapshot of the network and the average accuracy of the demographic gravitation "law" in capturing edge removals over the entire period of our data in Fig. S8b and S8c. Our machine learning approach produces better results than the demographic gravitation "law".

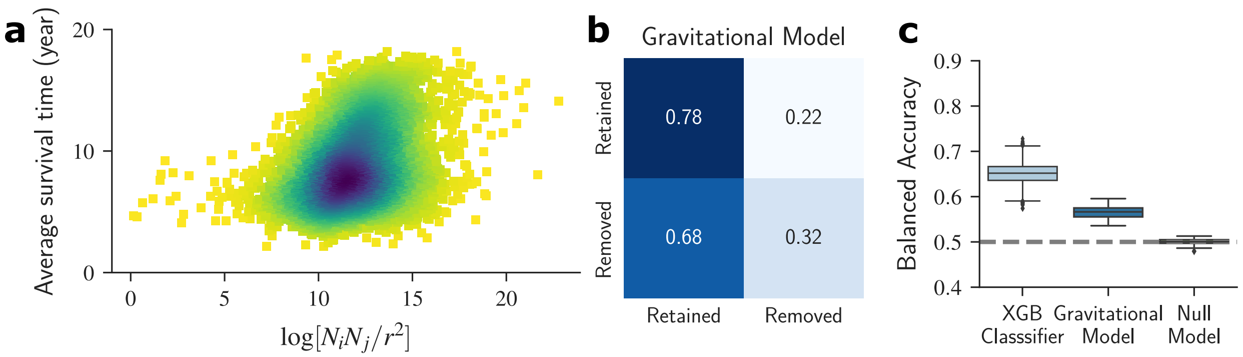

FIG. S8. **Results for removal based on inter-city flow predicted by the demographic gravitation "law".** **(a)** Edges predicted with longer average survival time have on average higher values of demographic gravitation flow, which has been proposed to approximate the human mobility between two regions. The correlation coefficient between the average survival time and the demographic gravitation flow is $\rho = 0.33$ (p−value $< 10^{-16}$). However, there is greater variation in the data, suggesting that our approach better captures the dynamics of the system. **(b)** Confusion matrix for the model prediction of one snapshot of the U.S. Air net. **(c)** The box plots show the balanced accuracies for non-simultaneous tests for all snapshots for the U.S. Air net.

[1] Pedregosa, F. *et al.* Scikit-learn: Machine learning in Python. *Journal of Machine Learning Research* **12**, 2825–2830 (2011).

[2] Chen, T. & Guestrin, C. XGBoost: A scalable tree boosting system. In *Proceedings of the 22nd ACM SIGKDD International Conference on Knowledge Discovery and Data Mining*, 785–794 (ACM, 2016).

[3] Newman, M. E. J. Analysis of weighted networks. *Phys. Rev. E* **70**, 056131 (2004).

[4] Ahmadi, A. A. & Zhang, J. On the complexity of finding a local minimizer of a quadratic function over a polytope. *arXiv preprint arXiv:2008.05558* (2020).

[5] Barthelemy, M. The statistical physics of cities. *Nature Reviews Physics* **1**, 406–415 (2019).

[6] United States Census Bureau. City and Town Population Totals: 2010-2019 (2019). URL `https://www.census.gov/`.
